# Full median sternotomy approach for treatment of upper thoracic vertebral tuberculosis in a developing country: case report and short literature review

**DOI:** 10.11604/pamj.2017.28.112.12948

**Published:** 2017-10-05

**Authors:** Isaac Okyere, Anthony Lamina, Benedict Owusu

**Affiliations:** 1 Cardiothoracic and Vascular Surgeon, Department of Surgery, School of Medical Sciences, Kwame Nkrumah University of Science and Technology and Komfo Anokye Teaching Hospital,Kumasi, Ghana; 2 Neurosurgeons, Department of Surgery, Komfo Anokye Teaching Hospital, Kumasi, Ghana

**Keywords:** Osteomyelitis, cervicothoracic junction, median Sternotomy, laminectomy

## Abstract

Direct anterior approach to the cervicothoracic spine (C7-T4) poses a technical challenge in neurosurgery, due to the presence of important neurovascular structures anterior to the cervicothoracic junction (CTJ). Median Sternotomy approach is a surgical option that allows for direct anterior exposure of the lower cervical and upper thoracic vertebrae. We report the first case from Ghana, West Africa of a young man who developed post-tuberculosis osteomyelitis of upper thoracic (T1-2) vertebrae with cord compression after spinal tuberculosis in childhood. He underwent a full median Sternotomy for Anterior Decompression and Fusion of C7-T2 with autologous iliac crest bone graft. We detail our operative procedure and review the relevant literature.

## Introduction

Obtaining direct ventral access to the cervicothoracic spine (C7-T2) for decompression and fusion is technically challenging, given the anatomical constraints. Operative exposure of the cervicothoracic junction (CTJ) is obscured by the skeleton of the thorax (i.e, sternum, clavicles and ribs). The anterior transsternal or median sternotomy approach is a feasible surgical option that allows for direct exposure of the anterior vertebral elements of the CTJ. With a transsternal approach, there are many vital neurovascular structures close by, so great care needs to be carried out while dissecting away the vascular compartment of the superior mediastinum to achieve adequate exposure. Anatomical structures at risk for injury include structures within the carotid sheath, trachea, esophagus, recurrent laryngeal nerves, great vessels, vertebral arteries and sympathetic trunk [1]. We report the first case from Ghana, West Africa of a young man who developed post-tuberculosis osteomyelitis of upper thoracic (T1-2) vertebrae after childhood spinal tuberculosis. He underwent a full median Sternotomy for ventral decompression and fusion of C7-T2.

## Patient and observation

Informed patient consent was obtained for this patient's treatment.

**Figure 1:**
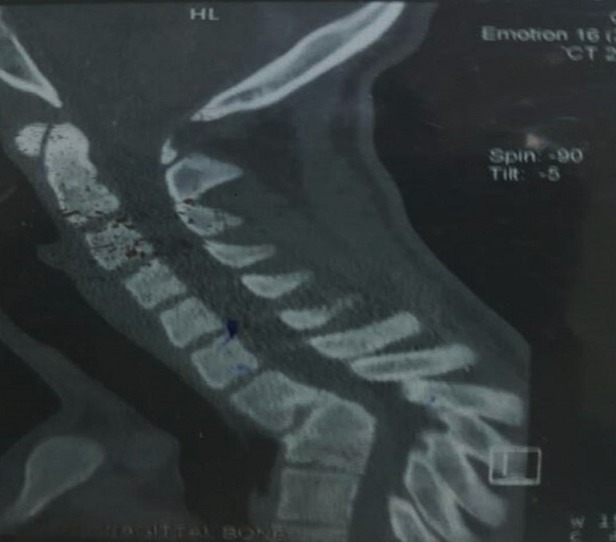
Preoperative CT Imaging with spinal cord compression at level T1/T2

**Figure 2:**
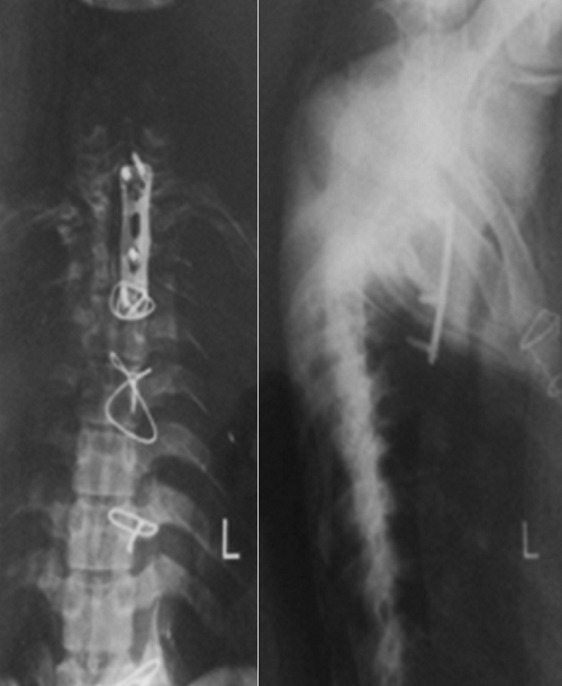
Anterior-posterior + lateral X-rays views

**Figure 3:**
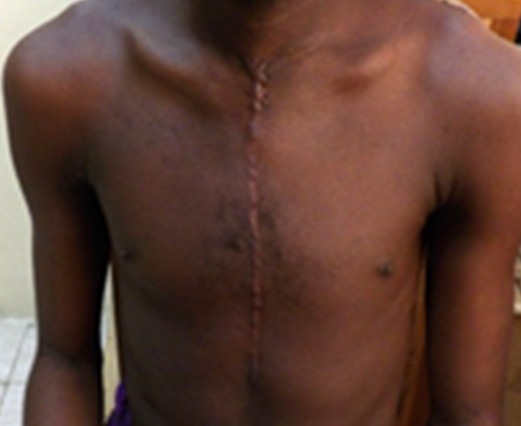
Sternotomy scar

**History**: The patient was a twenty (20) year old Senior High School graduate with a childhood history of spinal Tuberculosis who has had successful treatment and had been well until he started experiencing progressive weakness in all the limbs after graduating from Senior High School. This was associated with inability to get up from the sitting position and to grab things well. He had also noted loss of muscle weakness in the calf and thigh muscles but not in the upper limbs and could not climb stairs. On examination he had Gibbus at T1/T2, myoclonus and increased tendon reflexes bilaterally in the lower limbs and wasting of the calf muscles. Power was 4/5 in all limbs. CT Scan of the Cervical spine showed cervical kyphosis and reduced height and anterior wedge deformity of T1 and T2 vertebral bodies kinking the cord. Also noted was ankylosis of T1 and T2 vertebral bodies. No evidence of a significant paravertebral soft tissue mass was noted. Conclusion was a severe kyphotic deformity at T1/T2 and T1/T2 ankylosis as sequelae of previous TB infection with associated cord compression Figure 1, Figure 2, Figure 3.

**Operation**: The patient was placed supine on the operating table under general endotracheal anesthesia and the neck hyperextended and turned to the left side. A rolled towel was placed between the scapulae for better exposure after Sternotomy. A nasogastric tube was passed to enable better intraoperative identification of the esophagus and for suspected thoracic duct leakage. The patient was prepped from the neck to the midthigh and drapped appropriately. Prophylactic antibiotics was administered. Then we performed a full median Sternotomy with a sternal saw. Bleeding from the sternal plates was stopped with bone wax and a retractor inserted. The previous unsuccessful right collar incision by the neurosurgery team was extended down to the manubrium. The T1 and T2 lesions were localized after careful retraction of the vital neurovascular structures with the brachiocephalic trunk and right common carotid arteries being gently retracted to the right and the esophagus and trachea to the left, using self-retaining retractors. Fluoroscopy was utilized to identify the C7-T2 Vertebrae. There was kyphotic deformity at C7/T1/T2 and near total destruction of T1 vertebral body with residual bony fragment compressing the cord. Under fluoroscopy, the C6/C7 disc space was identified and conventional corpectomies of C7 and T1 using pituitary rongeurs and curettes were under taken. The thecal sac was freed up and the spinal cord from C7-T2 was decompressed. An anterior arthrodesis and fusion from C7-T2 was achieved with an autograft bone graft from the left iliac crest. A cervical titanium plate was drilled and tapped in place using 4 screws to stabilize the graft. The surgical site was thoroughly irrigated with Normal Saline. Hemostasis was achieved with bipolar cautery and vacuum drain was placed. The sternum was closed with No. 6 sternal wires and the incision was closed up in layers. There was no intraoperative complication and estimated blood loss (EBL) was 200 cc.

**Postoperative course**: The patient looks good four months after surgery. Postoperative x-rays look good and he is recovering muscle power and neurological derangement under intense physiotherapy and rehabilitation.

## Discussion

The median Sternotomy approach to the pathologies of the lower cervical and upper thoracic vertebrae really offers an adequate direct exposure option. Meticulous dissection of the anteriosuperior mediastinum with isolation and retraction of the vital neurovascular structures prior to operating on the spine is very important to avoid postoperative morbidity and mortality. Both Zengming et al [2] and Jiang et al (2010) [1] reported their experience with anterior decompression and fusion via Sternotomy. The report showed a resolved pain in all patients, improvement in motor deficits especially in those patients who had initially presented with radiculopathy or myelopathy and these findings correlates well with our patient who is currently pain free and is improving in his movement. These were associated with no postoperative approach-related complication. The transsternal approach presented in this article utilizes a full median Sternotomy for access of the cervicothoracic spine. However, there have been many modifications to this approach in order to limit extensive osteotomy, such as manubriotomy with clavicle resection, partial lateral. Manubriotomy and partial Sternotomy with a transverse sternal split [3-7]. There are several potential advantages of a full median Sternotomy approach. First of all, it is a technically simpler procedure compared to other modified approaches as experienced in our case, second, it offers better exposure of the mediastinum for improved visualization and manipulation of important neurovascular structures to avoid intraoperative complications. Third, the pectoral girdle is preserved since there is no resection of the clavicle. Lastly, extension of the operative field caudally to as low as T5 can be achieved by dissecting a plane between the brachiocephalic vein, superior vena cava and ascending aorta as performed for our case [1]. As far as we know, this is the first reported case from Ghana and West Africa.

## Conclusion

The anterior approach to directly access the pathologies of the lower cervical and upper thoracic vertebrae via a full Median Sternotomy is a safe and effective surgical approach option. Full Median Sternotomy is simpler, provides better exposure and it does not complicate the shoulder girdle. The incision can also be extended caudally and cephally.

## Competing interests

The authors declare no competing interests.
